# Cross-species evaluation of fibroblast activation protein alpha as potential imaging target for soft tissue sarcoma: a comparative immunohistochemical study in humans, dogs, and cats

**DOI:** 10.3389/fonc.2023.1210004

**Published:** 2023-09-01

**Authors:** Patricia Beer, Chantal Pauli, Martina Haberecker, Paula Grest, Erin Beebe, Daniel Fuchs, Enni Markkanen, Christiane Krudewig, Mirja Christine Nolff

**Affiliations:** ^1^ Clinic for Small Animal Surgery, Department for Small Animals, Vetsuisse Faculty, University of Zurich, Zurich, Switzerland; ^2^ Department of Pathology and Molecular Pathology, University of Zurich, Zurich, Switzerland; ^3^ Medical Faculty, University of Zurich, Zurich, Switzerland; ^4^ Institute of Veterinary Pathology, Vetsuisse Faculty, University of Zurich, Zurich, Switzerland; ^5^ Institute of Veterinary Pharmacology and Toxicology, Vetsuisse Faculty, University of Zurich, Zurich, Switzerland

**Keywords:** comparative oncology, animal models, TASC-score, molecular imaging, biomarker, fluorescence guided surgery, digital pathology

## Abstract

**Introduction:**

Complete surgical tumor resection is paramount in the management of soft tissue sarcoma (STS) in humans, dogs, and cats alike. Near-infrared targeted tracers for fluorescence-guided surgery (FGS) could facilitate intraoperative visualization of the tumor and improve resection accuracy. Target identification is complicated in STS due to the rarity and heterogeneity of the disease. This study aims to validate the expression of fibroblast activation protein alpha (FAP) in selected human, canine, and feline STS subtypes to assess the value of FAP as a target for FGS and to validate companion animals as a translational model.

**Methods:**

Formalin-fixed and paraffin-embedded tissue samples from 53 canine STSs (perivascular wall tumor (PWT), canine fibrosarcoma (cFS), and STS not further specified (NOS)), 24 feline fibrosarcomas, and 39 human STSs (myxofibrosarcoma, undifferentiated pleomorphic sarcoma, dermatofibrosarcoma protuberans, and malignant peripheral nerve sheath tumor) as well as six canine and seven feline healthy controls and 10 inflamed tissue samples were immunohistochemically stained for their FAP expression. FAP labeling in tumor, peritumoral, healthy skin, and inflamed tissue samples was quantified using a visually assessed semiquantitative expression score and digital image analysis. Target selection criteria (TASC) scoring was subsequently performed as previously described.

**Results:**

Eighty-five percent (85%) of human (33/39), 76% of canine (40/53), and 92% of feline (22/24) STSs showed FAP positivity in over 10% of the tumor cells. A high expression was determined in 53% canine (28/53), 67% feline (16/24), and 44% human STSs (17/39). The average FAP-labeled area of canine, feline, and human STSs was 31%, 33%, and 42%, respectively (*p* > 0.8990). The FAP-positive tumor area was larger in STS compared to healthy and peritumoral tissue samples (*p* < 0.0001). TASC scores were above 18 for all feline and human STS subtypes and canine PWTs but not for canine STS NOS and cFS.

**Conclusion:**

This study represents the first cross-species target evaluation of FAP for STS. Our results demonstrate that FAP expression is increased in various STS subtypes compared to non-cancerous tissues across species, thereby validating dogs and cats as suitable animal models. Based on a TASC score, FAP could be considered a target for FGS.

## Introduction

Soft tissue sarcomas (STSs) are mesenchymal tumors consisting of a variety of histological and molecular different subtypes that affect humans as well as companion animals (1, 2). In adolescents, STSs belong to the group of so-called rare cancers comprising less than 1% of all malignant tumors, with incidence rates ranging between 4 and 7 per 100,000 people per year (3, 4). In dogs, STSs are 10 times more common, representing 10.9% of all diagnosed malignancies with an incidence rate of 40.1 per 100,000 dogs per year (5). Over 70 different human STS subtypes can be identified (6); among these, undifferentiated pleomorphic sarcoma (UPS), liposarcoma, myxofibrosarcoma (MFS), and malignant peripheral nerve sheath tumor (MPNST) are common subtypes (4, 7). The classification of STS on the sole basis of conventional microscopic morphology is complicated, and immunohistochemical (IHC) and molecular analyses are indispensable (6). In companion animals, differentiation is not as distinct, and much less subtypes have been described (8). Common diagnosed STS entities in dogs are perivascular wall tumors (PWTs), canine fibrosarcoma (cFS), or STS not further specified (STS NOS) (5). In the feline population, STS represents the second most common skin tumor, making up 17% of tumor diagnoses in this species, and feline fibrosarcoma (fFS) is the most common subtype (9, 10).

Irrespective of the actual tumor entity, histologic types, tumor grades, and the biological behavior of STS are comparable across species with often a dismal outcome (2, 11). Treatment of STS is interdisciplinary, and a case-specific evaluation is mandatory in order to decide which treatments must be applied (1). While adjuvant or neo-adjuvant radiation therapy (RT) and chemotherapy are important pillars of a tailored therapeutic plan, first-line treatment for localized and resectable STS remains complete tumor resection in all species (1, 8, 12, 13). STS displays a high tendency for local recurrences due to their aggressive local behavior (11, 14–19). The success of surgical procedures is essential, as complete resection remains the only important prognostic factor for local recurrence and disease progression that clinicians can influence in all species (20–22). Although preoperative functional and/or molecular imaging modalities are increasingly used to improve perioperative planning (23, 24), precise estimation of the true tumor margin during surgery can remain very challenging. The presence of not clinically visible cellular tumor extensions infiltrating the peritumoral tissue is an obstacle that the surgeon cannot overcome by vision or tactile investigation (8, 12). Even following resection with wide margins, incomplete margins after resection occur in up to 28% of human patients and up to 41% of dogs, increasing the risk for local recurrence and disease progression (12, 21, 25). In humans, the completeness of surgery is highly dependent on the preoperative planning and the expertise of the institution. Treatment in specialized referral centers with a high number of selected patients is therefore highly recommended. Improved tumor visualization in the operating room represents an unmet clinical need, and the development of new imaging approaches bridging the gap between preoperative radiologic imaging and postoperative histopathological assessment remains an area of active investigation in this disease to address this shortcoming (25–28).

Near-infrared (NIR) fluorescence-guided surgery (FGS) introduces a new approach to address this challenge in surgical oncology. A targeted fluorescent contrast agent is injected intravenously and accumulates in the tumor tissue where it emits light in the NIR spectrum (650–800 nm) after excitation with specific NIR camera systems (28). For specific accumulation of the fluorescent dye in the tumor, a fluorophore is coupled to a targeting moiety—a small molecule, peptide, or antibody—that specifically recognizes a target overexpressed in the tumor cells or the tumor microenvironment (26). The enhanced NIR signal in the cancerous tissue can then guide the surgeon toward a more precise tumor resection. Proper identification of tumor-specific targets for molecular imaging is key to the success of this technique (25). Although the technique of targeted NIR-based tumor imaging is rapidly evolving (25, 27, 29) and the first targeted dye has been approved by the Food and Drug Administration (FDA), the heterogeneity and low incidence of STS make identification of targets challenging. So far, only one phase I clinical study evaluating bevacizumab-800CW, a NIR dye targeting vascular endothelial growth factor, is available in human patients with different types of STS. This study documented acceptable *in vivo* safety and general feasibility (30).

Recently, fibroblast activation protein alpha (FAP) has gained interest as a potential target in solid tumors. FAP is a cell surface glycoprotein with dipeptidyl peptidase and endopeptidase activity (31). It is known to be primarily expressed in cancer-associated fibroblasts (CAFs), the major cell component in the tumor microenvironment of solid tumors. CAFs are key players in tumor progression, confer treatment resistance, and promote tumor invasion, metastasis formation, and immunosuppression (32). Epithelial tumor cells and fibroblasts in benign conditions do not typically express FAP, while expression of FAP on mesenchymal tumor cells was documented by IHC in two human studies (33, 34). FAP overexpression in CAFs and cancer cells is also associated with a worse prognosis (35). FAP inhibitors (FAPIs) have been radiolabeled for the purpose of molecular imaging using PET/CT, and a recent study in adults with STS documented the feasibility to image STS using this target (24). Finally, FAP-targeted radioligand therapies are currently under investigation (36).

Based on these results, it seems reasonable to investigate the expression of FAP across different STS entities in different species to evaluate if i) FAP might be a potential target for future NIR imaging and ii) expression is comparable between species to determine the translational value of dogs and cats as models.

The aim of this study was, therefore, to assess the expression of FAP in formalin-fixed and paraffin-embedded (FFPE) human, canine, and feline STS samples as well as in inflammatory and normal healthy tissue samples using IHC and to determine the target selection criteria (TASC) score (37, 38).

## Materials and methods

### Selection of canine and feline tissues

FFPE archival tissue samples of canine and feline STSs were retrieved from the archives of the Institute for Veterinary Pathology, University of Zurich. STS samples comprised biopsies or surgical resections with the diagnosis of canine PWT, cFS, canine STS NOS, and fFS. Diagnosis and grading were performed for clinical purposes by two board-certified pathologists (P.G. and C.K.) using 2-μm sections stained with a routine hematoxylin and eosin (H&E) stain and IHC. Diagnoses of sarcomas in dogs and cats were made according to the current guidelines proposed by the Davis-Thompson Foundation (39). Periaxin and ionized calcium-binding adapter protein 1 staining were performed in canine STS to rule out peripheral nerve sheet tumors and histiocytic sarcoma. Feline FS occurred predominantly at injection sites and also included tumors with characteristic features of injection-site sarcoma.

Healthy control samples including skin, subcutis, and muscle were harvested from six dogs and seven cats less than 6 hours after euthanasia due to reasons not associated with this study. The tissue samples were fixed in 10% neutral buffered formalin for 24 hours to 48 hours and thereafter routinely processed and embedded in paraffin. Routine H&E staining was performed to exclude any underlying skin and muscle pathology. Archived FFPE tissue samples from dermal and subcutaneous inflammatory lesions of 10 dogs and 10 cats were used for comparison with FAP expression in STS.

### Selection of human tissue

Human FFPE tumor tissue samples were retrieved from the archives of the Department of Pathology and Molecular Pathology, University Hospital Zurich. The use of tissue samples derived from humans was approved by the Cantonal Ethics Commission of Zurich (BASEC-2021-00417). STS subtypes used for this study were MFS, UPS, dermatofibrosarcoma protuberans (DFSP), and MPNST and had been diagnosed and graded for clinical purposes by an expert soft tissue and bone pathologist (C.P.) according to WHO guidelines. Two-micrometer sections were cut, and a routine H&E stain was performed to define tumor areas and margins as well as healthy adjacent tissues.

The use of tissue samples derived from humans was approved by the Cantonal Ethics Commission of Zurich (BASEC-2021-00417). The use of canine and feline tissue samples from archives does not require ethical approval.

### Anti-FAP immunohistochemistry

Immunohistochemistry was performed using a primary anti-FAP alpha recombinant monoclonal rabbit antibody (ab207178; EPR20021; Abcam, Cambridge, UK). Antibody validation for canine and feline was demonstrated by Western blotting (**Supplementary Material**). Control tissue samples for IHC in dogs and cats comprised a canine STS and a feline mammary carcinoma, and for humans, the sample comprised breast cancer. As negative control tissue in dogs and cats, a healthy skin sample was used, and in humans, dogs, and cats, the primary antibody was omitted from the positive control. Staining protocols were established and optimized for each species. The primary anti-FAP alpha antibody dilution was chosen after assessment of the dilution series in each species, determining a concentration with a high signal and low background staining: 1:200 (canine tissue), 1:150 (feline tissue), and 1:100 dilution (human tissue).

Of FFPE canine and feline tissue blocks, 2-μm-thick sections were mounted on positively charged slides (SuperFrost Plus slides, Thermo Fisher Scientific, Waltham, MA, USA) and dried overnight at 37°C. Unstained sections were deparaffinized with four xylene baths for 5 min each using the Tissue-Tek Film (Sysmex, Kobe, Japan) followed by rehydration using degressive alcohol series (100%, 95%, and 70% ethanol) and rinsing in distilled water. Slides were incubated in ethylenediaminetetraacetic acid buffer (pH 9.0) for 20 minutes in a pressure cooker set to 98°C for heat-induced antigen retrieval, followed by a washing step in distilled water. Slides were put into Tris-buffered saline wash buffer (Dako, Carpinteria, CA, USA; 3006) before being stained in the Dako Autostainer. The primary antibody was diluted, and incubation was performed for 60 min at room temperature for canine tissue and overnight for feline tissue at 4°C, followed by peroxidase blocking (peroxidase blocking buffer, Dako S2023) for 10 min at room temperature and incubation with the secondary antibody (Envision+System HRP Rabbit (Dako K4003)) for 30 min at room temperature. Between those steps, slides were rinsed with Tris-buffered saline wash buffer (Dako 3006). For visualization, the chromogen diaminobenzidine (DAB Detection Kit (Dako K3468)) was used with an incubation time of 10 min at room temperature followed by rinsing with distilled water. All sections were counterstained with hematoxylin for 2 seconds, rinsed with tap water, dehydrated in the Prisam (Sysmex) with increasing Xylol series (70%, 95%, and 100% Xylol), and coverslipped with the Tissue-Tek Film. Human tissue staining was performed using the Bond LeicaRX staining platform. Tissue was pretreated using an epitope retrieval solution buffer H2 Leica applied at 100°C for 30 minutes. The primary antibody (ab207178) was used as a 1:100 Bond Antibody Diluent and applied for 30 min. For visualization, Bond Polymer Refine Detection Kit DS9800 was used.

### Qualitative and semiquantitative analyses of FAP expression

The qualitative and semiquantitative analyses of human tissue samples were performed by two pathologists (C.P. and M.H.) with 8 and 5 years of experience in this field, respectively. Canine and feline tissue assessments were performed by a board-certified veterinary pathologist (C.K.) with 23 years of experience and a trained PhD student (P.B.) under the supervision of a veterinary pathologist. The evaluation was performed on digitalized slides and included the type of labeled cells (stromal cells and tumor cells), uniformity of staining in FAP-positive cells (pattern of expression), staining homogeneity throughout the tumor sample, percentage of stained tumor area, and intensity of staining as previously described (40). The overall percentage of FAP tumor labeling was scored as previously described: +1, absence of or weak FAP labeling in <1% of the cells; 2+, labeling of 1% to 10% of the cells; 3+, labeling of 11% to 50% of the cells; and 4+, labeling of over 50% of tumor cells. labeling intensity was graded as follows: 0, no labeling; 1, weak labeling; 2, intermediate labeling; and 3, strong labeling (40).

**Figure 1:**
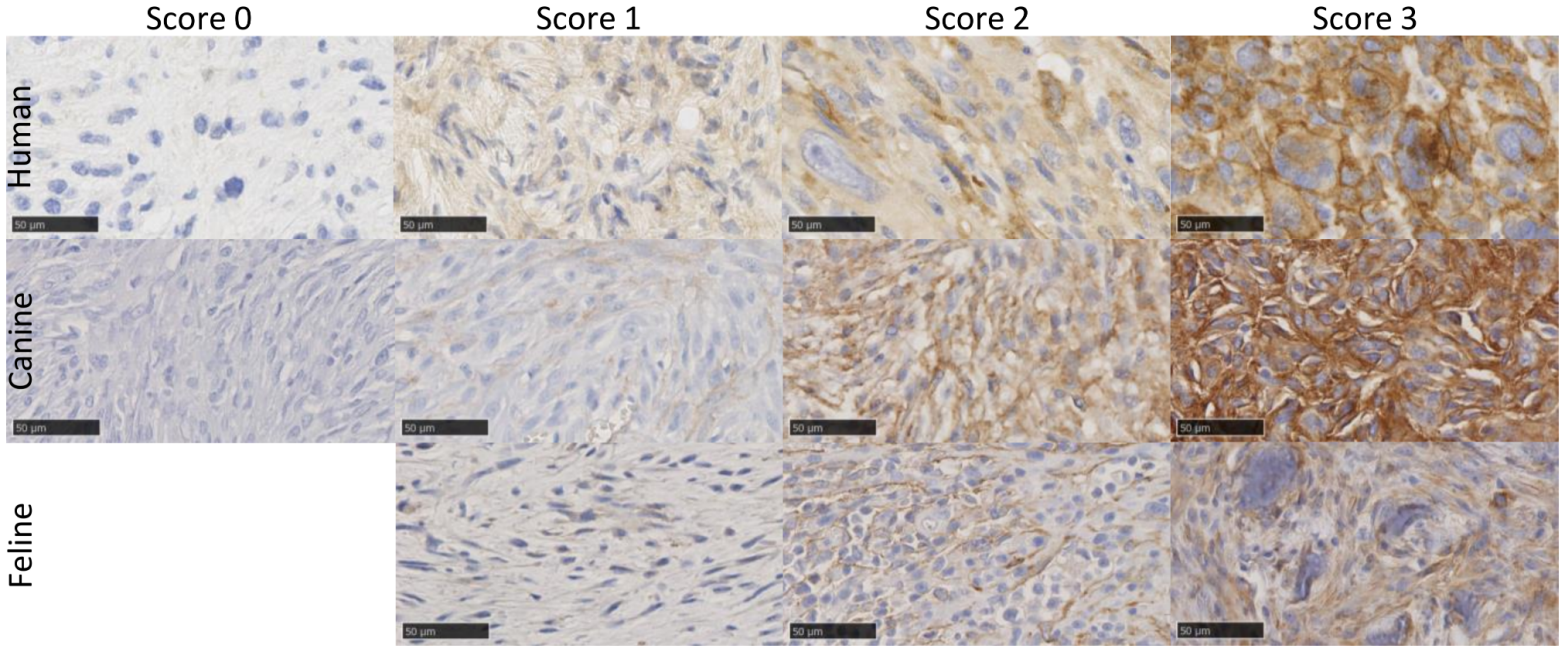
Example images of FAP-stained human, canine, and feline soft tissue sarcomas using a monoclonal anti-FAP antibody (dilution 1:100 human, 1:150 feline, and 1:200 canine). Tissue samples showed varying FAP expression, which was graded using a semiquantitative FAP expression score ranging from score 0 (no expression), score 1 (low expression), score 2 (intermediate expression) to score 3 (high expression). In cats, all tumors showed at least a low FAP positivity and had a score of 1 or higher, and no score of 0 was given. The images show examples of cytoplasmic (e.g., human score 2 and feline score 3) and membranous (e.g., human score 3 and canine score 3) staining patterns of tumor cells. Scale bar 50 µm. FAP, fibroblast activation protein alpha.

A final grade of positivity was calculated by multiplying both scores. A final expression score was considered as no expression at a value of 0, low at 1 to 3, intermediate at 4 to 6, and high at 8 to 12 (**Figure 1**). If a tumor sample had an inhomogeneous staining pattern with more than one-third of the tumor area showing a higher staining intensity, evaluation was performed in areas with a strong IHC signal.

### Quantitative image analysis

**Figure 2:**
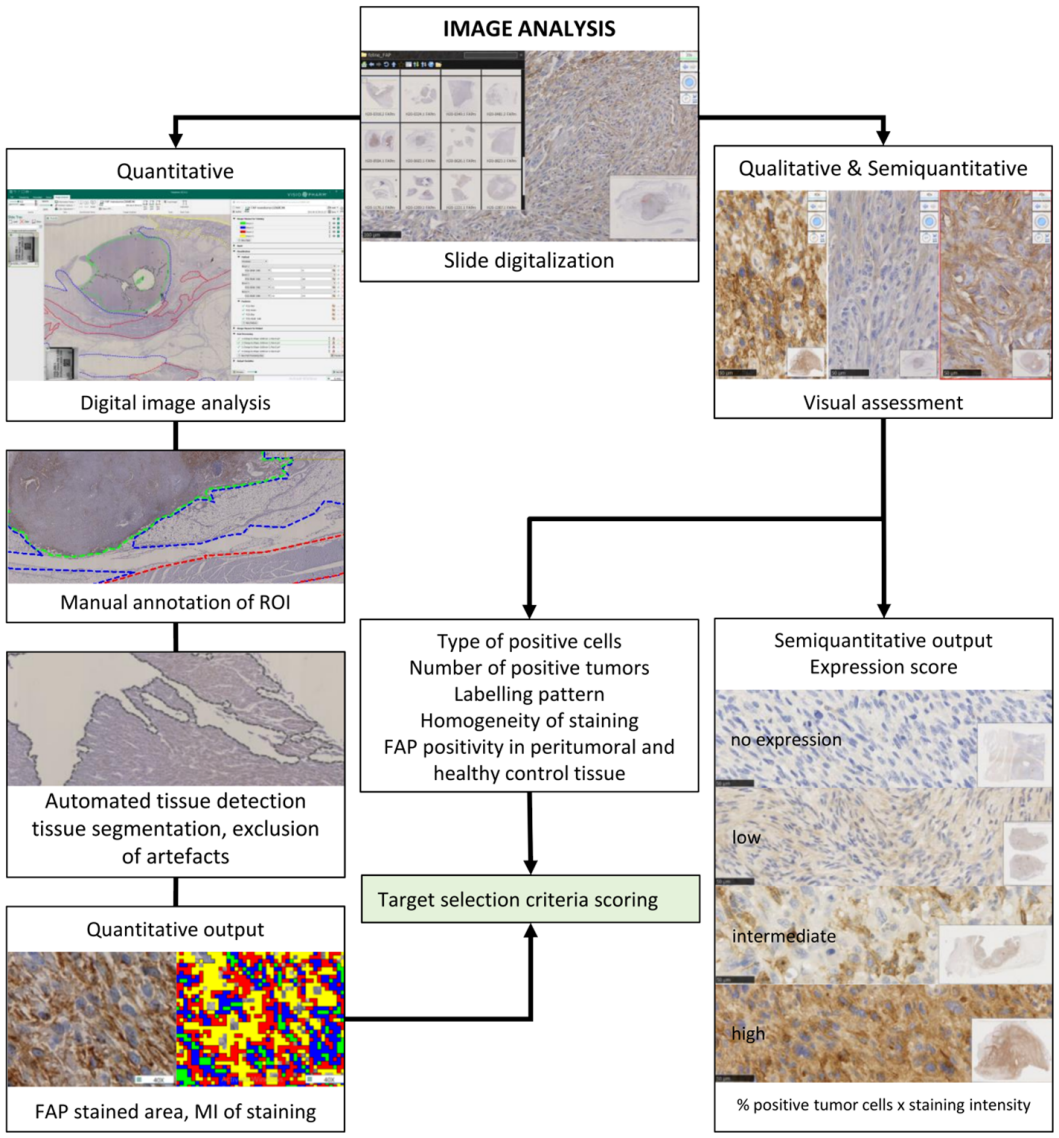
Schematic representation of the strategy for visual and digital image analyses. Anti-fibroblast activation protein alpha and routine hematoxylin and eosin-stained glass slides were scanned in a Hamamatsu NanoZoomer 2.0-HT scanner. They were used for quantitative, semiquantitative, and qualitative image analyses. A qualitative visual assessment of FAP-labeled tissue was performed by pathologists, and a semiquantitative FAP expression score was defined for each tumor. For automated quantitative image analysis using artificial intelligence, digitized slides were stored on a centralized server, and a direct link was established with the Visiopharm Integrator System (VIS) version 6.9.0.2779 (Visiopharm, Hørsholm, Denmark) platform. The first step of digital image analysis was the manual delineation of the regions of interest (ROIs). Each color represents one tissue type that was assessed separately. Automated tissue detection for segmentation and exclusion of artifacts (in gray) was performed. Automated visual detection and quantification of FAP labeling resulted in quantitative output measures. The algorithm for the different brown groups was designed so that it uses a pixel intensity thresholding for the level 1 (FAP-positive cells) weak immunoreactivity of 131–175 pixels; level 2, intermediate immunoreactivity (101–130 pixels); level 3, strong immunoreactivity (71–100 pixel); level 4, very strong immunoreactivity (<71 pixels). Pixel values above 176 were considered as background staining intensity (FAP-negative cells). The FAP-labeled areas, the staining intensity levels, and the mean staining intensity were used for statistical comparison. Results of the qualitative and quantitative FAP tumor labeling were used for target selection criteria scoring. FAP, fibroblast activation protein alpha.

An automated histomorphometry software Visiopharm® (Hørsholm, Denmark) was used for the quantification of FAP staining in digitalized whole-slide images (**Figure 2**). The first annotation of regions of interest (ROIs) was performed roughly manually (P.B.) before the software detected and excluded artifacts from these ROIs. The ROIs were set to delineate tumor, peritumoral, and control tissues separately. For peritumoral and control to tissue, three different ROIs were chosen: the epidermis and dermis (collagen) and adipose and muscle tissues. In the inflammatory controls, the inflamed areas were included in the ROI (inflammation) and distinguished from adjacent non-inflammatory tissues.

**Figure 3:**
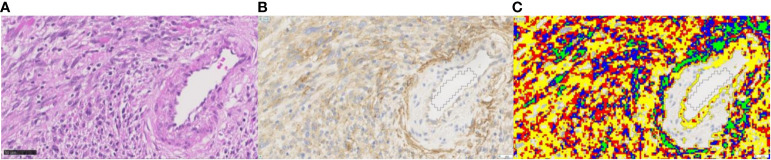
Example for quantitative image analysis of FAP staining in grade 3 human malignant peripheral nerve sheath tumor. **(A)** The H&E-stained section, **(B)** the FAP IHC section, and **(C)** the corresponding FAP-positive areas after quantification in color. Different colors represent the different labeling intensity levels ranging from level 1 (yellow; weak immunoreactivity) to level 2 (red; intermediate), level 3 (blue; strong), to level 4 (green; very strong immunoreactivity). The FAP-positive tumor cells show a cytoplasmic staining pattern of intermediate staining intensity. Endothelial cells of the artery on the right side of the image show a weak positivity for FAP, while the smooth muscle cells are negative. Myofibroblasts of the tunica adventitia show strong FAP positivity. Magnification ×40. FAP, fibroblast activation protein alpha.

Within the second step, for each tissue type, one application was designed to measure the FAP-labeled area and the mean intensity (MI) of staining (**Figure 3**). The FAP-labeled area was stratified by the intensity of staining into areas of five levels (0–4), ranging from background staining (negative) to weak, intermediate, strong, and very strong immunoreactivity (**Figure 3**). The total FAP positively stained area was calculated as the sum of levels 1–4 for each slide. Due to the different sizes of ROIs, all measured areas within these ROIs were normalized to a standardized area of 10 high-power (×400) fields (HPF) for all further comparisons (2.37 mm^2^ in total) (41). The second output measure was defined as the overall MI of the ROI expressed as pixel value with lower values representing a stronger brown staining for FAP.

### Target selection criteria scoring

In order to evaluate the potential for future imaging, the TASC score was applied as previously described (37, 38) (**Table 1**). Diffuse and focal overexpression of FAP in STS samples (criterion II) was semiquantitatively assessed by estimating the percentage of positive tumor cells. The assessment was performed by two investigators for canine and feline tumors (P.B. and C.K.) and two pathologists for human tissue samples (M.H. and C.P.). The number of positive STS samples with more than 50% positive tumor cells was then calculated. The tumor-to-normal (T/N) tissue ratio of FAP was calculated using the results of the quantitative IHC analysis. The standardized areas of FAP labeling (intensity levels 1–4) for each STS sample were compared with the standardized areas of FAP labeling in non-neoplastic tissues. An average of the labeled area in the peritumoral and healthy control tissue samples was calculated for each species and tumor entity including muscle, adipose, and epidermis/dermis tissues. For every single tumor, the ratio between the FAP-labeled tumor area to the average FAP-labeled control tissue area was calculated. To receive a T/N ratio for the different species and STS entities, the number of tumors with a ratio under and over 10 was calculated within the different groups. The ratio of a group was judged as >10 if the majority (>50%) of the tumors had a T/N ratio greater than 10.

**Table 1:** Description of the target selection criteria scoring (TASC) published by van Oosten et al. (2011) (38).

Criteria		Description of the scoring system	Maximum score	FAP scoring
I	Extracellular protein localization	Receptor bound to cell surface = 5 In close proximity to the tumor cell = 3	5	5 (31)
II	Diffuse overexpression through tumor tissue	Staining ≥50% of tumor cells in the majority (>50%) of patients = 4 Staining <50% of tumor cells and/or in <50% of patients = 0	4	NA for STS
III	Tumor-to-normal tissue ratio	T/N > 10 = 3 T/N ≤ 10 = 0	3	NA for STS
IV	Percentage of overexpression in patients	≥90% = 6, 70%–89% = 5, 50%–69% = 3, 10%–49% = 0	6	NA for STS
V	Previous imaging success *in vivo*	Yes = 2 (including NIR imaging, PET/CT, MRI, and other imaging modalities)	2	2 (24, 42–44)
VI	Enzymatic activity	Yes = 1	1	1 (44–46)
VII	Target-mediated internalization	Yes = 1	1	1 (47–49)
**Total**			**22**	**≥9**

T/N, tumor-to-normal tissue ratio; PET/CT, positron emission tomography/computed tomography; MRI, magnetic resonance imaging; FAP, fibroblast activation protein alpha; STS, soft tissue sarcoma.

The percentage of tumors with overexpression of FAP was assessed by judging the FAP positivity for every single tumor and adding up the number of FAP-positive tumors. An STS was judged as positive if 10% of the tumor cells expressed an intermediate-to-strong staining pattern.

### Statistical analysis

Statistical analysis was performed using the commercially available software GraphPad Prism 9.1.2., La Jolla, CA, USA) and SPSS 27.0 statistical software (IBM Corporation, Armonk, NY, USA). Data were assessed for normal distribution. A non-parametric test for unpaired samples (Kruskal–Wallis test) was used to compare means of FAP-stained areas and MI pixel values between tumor, peritumoral, and control tissues, between STS subtypes, and between species. Dunn’s *post-hoc* correction was carried out on each pair of groups to adjust the *p*-value for multiple testing. For statistical comparison of categorical data (final grade of positivity), Pearson’s chi-squared test was used. Spearman’s coefficient was used to assess the correlation between FAP-stained area or MI pixel values and the tumor grade or final grade of positivity. *p* < 0.05 was considered statistically significant.

## Results

The included 53 canine STSs comprised 30 PWT, 16 STS NOS, and seven cFS (peritumoral tissue was available in 44 of these 53 samples). Feline STSs were 22 fFSs, all containing peritumoral tissue, and the 39 human STS entities included 10 MPNST, 10 UPS, 9 DFSP, and 10 MFS (peritumoral tissue available in 34 samples). Tumor grades are provided in **Table 2**.

**Table 2:** Grading of the included canine, feline, and human soft tissue sarcomas.

Tumor grade	Canine			Feline	Human			
	PWT (n = 30)	STS NOS (n = 16)	cFS (n = 7)	fFS (n = 24)	MPNST (n = 10)	UPS (n = 10)	DFSP (n = 9)	MFS (n = 10)
**Grade 1**	21 (70%)	8 (50%)	4 (57%)	5 (21%)	0	0	N/A	0
**Grade 2**	8 (27%)	7 (44%)	2 (29%)	17 (71%)	2 (20%)	0	N/A	0
**Grade 3**	1 (3%)	1 (6%)	1 (14%)	2 (8%)	7 (70%)	10 (100%)	N/A	10 (100%)

STS, soft tissue sarcoma; MPNST, malignant peripheral nerve sheath tumor; UPS, undifferentiated pleomorphic sarcoma; DFSP, dermatofibrosarcoma protuberans; MFS, myxofibrosarcoma; PWT, perivascular wall tumor; cFS, canine fibrosarcoma; STS NOS, STS not further specified; fFS, feline fibrosarcoma.

### Qualitative and semiquantitative visual assessments of FAP staining

Thirty-three human (33/39, 85%), 40 canine (40/53, 76%), and 22 feline (22/24, 92%) STSs showed FAP expression in over 10% of the tumor cells. The visually estimated mean percentage of FAP-labeled tumor cells was 51.1% ± 36.2% for canine, 56.6% ± 21.3% for feline, and 61.1% ± 41.7% for human STS (**Table 3**). While the FAP expression of tumor cells was primarily cytoplasmic in human STS, a more mixed expression in cytoplasm and cell membrane was apparent in canine and feline tumor cells (**Table 3**, **Figure 1**). There was a marked intertumoral heterogeneity of the FAP-positive areas and the intensity of staining ranging from strong positivity to complete absence of staining, in half of the STS tissue samples. **Table 4** lists the FAP positivity of different cell types of healthy, inflamed, and tumor tissue samples of human, canine, and feline tissue samples.

**Table 3:** Results of the qualitative and semiquantitative visual assessment of FAP tumor staining.

Parameter		Canine	Feline	Human
**Percentage of FAP-stained tumor cells (mean ± SD)**		51.1% ± 36.2%	56.6% ± 21.3%	61.1% ± 41.7%
**Labeling pattern**	Cytoplasmic	6/53 (11.3%)	1/24 (4.2%)	19/39 (48.7%)
	Membranous	3/53 (5.7%)	1/24 (4.2%)	1/39 (2.6%)
	Cytopl-membr	37/53 (69.8%)	21/24 (87.5%)	9/39 (23.1%)
	No expression	7/53 (13.2%)	1/24 (4.2%)	10/39 (39%)
**Staining of cells between or close to tumor cells**	Yes	28/53 (52.8%)	20/24 (83.3%)	31/24 (79.5%)
	No	25/53 (47.2%)	4/34 (16.7%)	8/24 (20.5%)
**Homogeneity of FAP labeling intensity throughout the tumor**	Homogeneous	33/53 (62.3%)	10/24 (41.7%)	23/39 (59%)
	Heterogeneous	20/53 (37.7%)	12/24 (50%)	16/39 (41%)
**Homogeneity of FAP-labeled cells throughout the tumor**	Homogeneous	33/53 (62.3%)	12/24 (50%)	18/39 (53.8%)
	Heterogeneous	20/53 (37.7%)	11/24 (45.8%)	21/39 (46.2%)

FAP, fibroblast activation protein alpha.

**Table 4:** Overview of the FAP-positive cell types in the different tissue types for the different species.

Tissue type	Species	Fibrocytes	Fibroblasts	Adipocytes	Endothelial cells	Peripheral nerve fibers	Muscle fibers	Immune cells
**Tumor tissue**	Human	Neg	Pos	Neg	Pos	Pos	Neg	Variable
	Canine	Neg	Pos	Neg	Pos	Pos	Neg	Variable
	Feline	Neg	Pos	Neg	Pos	Pos	Neg	Variable
**Inflamed tissue**	Canine	Neg	Pos	Neg	Pos	Pos	Neg	Variable
	Feline	Neg	Pos	Neg	Pos	Pos	Neg	Variable
**Healthy tissue**	Canine	Neg	n.a	Neg	Pos	Pos	Neg	n.a
	Feline	Neg	n.a	Neg	Pos	Pos	Neg	n.a

n.a., not applicable; FAP, fibroblast activation protein alpha.

**Figure 4:**
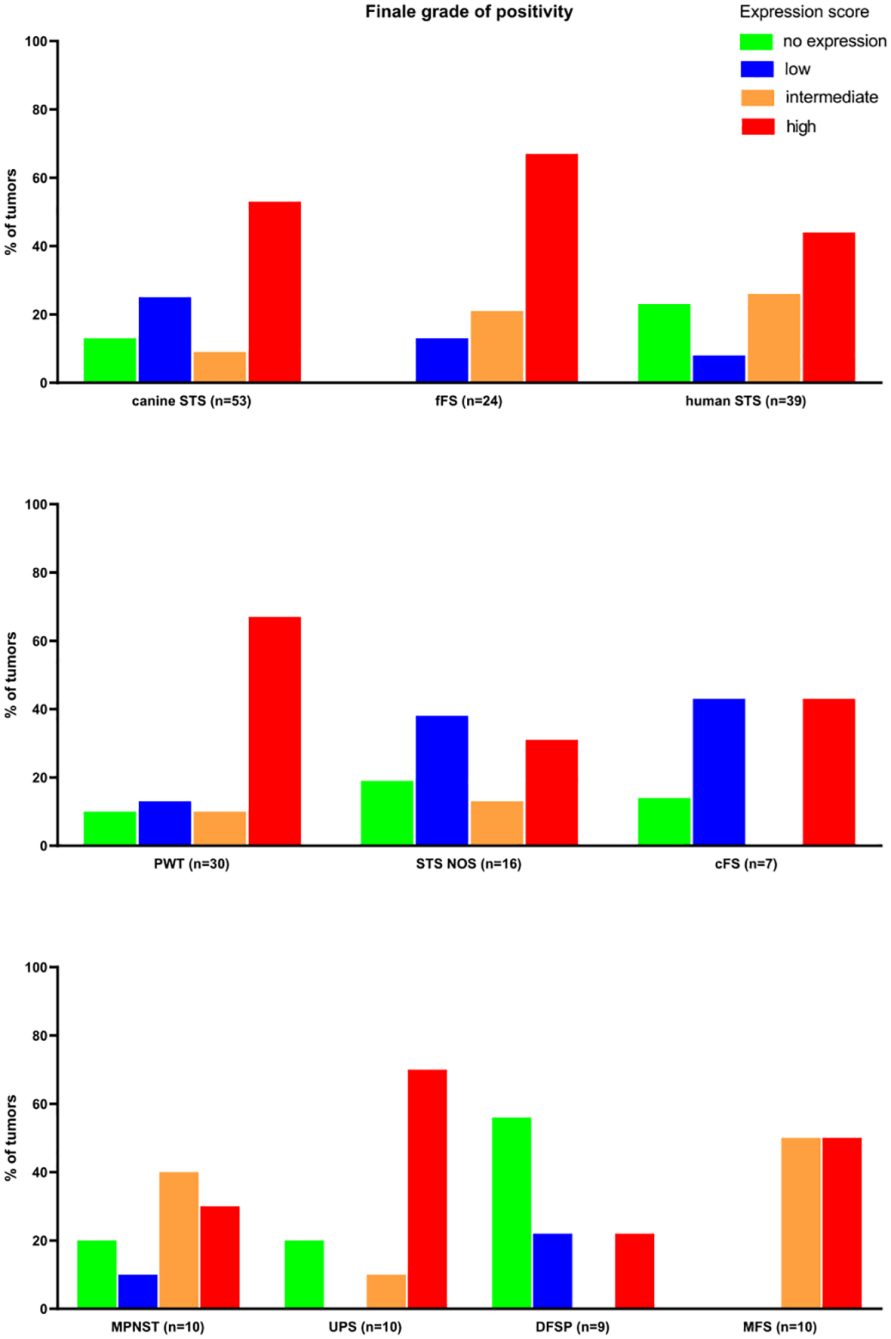
Results of the semiquantitative FAP expression scores. MPNST, malignant peripheral nerve sheath tumor; UPS, undifferentiated pleomorphic sarcoma; DFSP, dermatofibrosarcoma protuberans; MFS, myxofibrosarcoma; PWT, perivascular wall tumor; cFS, canine fibrosarcoma; STS NOS, soft tissue sarcoma not further specified; fFS, feline fibrosarcoma; FAP, fibroblast activation protein alpha.

Overall, the FAP expression score was high in 52.8% of canine (28/53), 66.7% of feline (16/24), and 43.6% of human STSs (17/39) (**Figure 4**). The highest expression scores were reached by PWT with canine STS and UPS within human STS entities. While all feline FSs reached at least a low FAP expression score, 13.2% of canine (7/53) and 23.1% of human STSs (9/39) were not expressing FAP. Most of the human STSs without FAP expression were DFSP (5/9). Taking together STSs with intermediate and high FAP expression scores, more than half of all tumors showed an intermediate-to-high FAP expression (canine STS, 33/53, 62.3%; feline STS, 21/24, 87.5%; human STS, 27/39, 69.2%).

### Quantitative assessment of FAP staining

The area of FAP-positive tumor tissue was similar in all species (*p* > 0.8990) with a relative stained area of 30.6%, 33.0%, and 42.2% in canine, feline, and human STSs, respectively. In all species, the FAP-positive area was significantly larger in the tumor tissue compared to peritumoral tissue of the same species (*p* < 0.0001) and in dogs and cats of the healthy control tissue (*p* < 0.0001), while no differences were detected comparing tumor tissue samples to inflamed canine and feline tissue samples (*p* > 0.9999). In cats, the FAP-stained area was larger in inflamed tissue samples compared to healthy feline control (*p* = 0.0001) and peritumoral tissue samples (*p* = 0.0063). No statistically significant differences were detected for the corresponding canine tissue samples. The FAP-stained area in peritumoral tissue samples was similar compared to healthy control tissue samples in dogs and cats (*p* > 0.2102). Dividing the FAP-stained area into the different staining intensity levels, the largest area of the tumors showed a high intensity of staining (level 4) in all species. No differences between species in the proportion of FAP-positive area were detected between the different intensity levels (*p* > 0.1634). For the MI pixel values, the lowest mean values, representing a more intense brown staining, were measured for human STSs followed by canine and feline STSs, with no significant differences (*p* > 0.4689). The mean staining intensity values were lower in tumor samples compared to peritumoral tissue samples of all species (*p* = 0.0127) and lower in tumor samples compared to the healthy control tissue samples in dogs and cats (*p* < 0.0011). There was no difference in the MI pixel values between tumor samples and inflamed tissue samples in canine and feline tissue samples (*p* > 0.9999). Likewise, the MI pixel values in canine and feline healthy control tissue samples were similar compared to the staining intensity in peritumoral tissue (*p* = 0.9999). Compared to healthy control tissue samples, the MI values measured in inflamed tissue samples of cats were lower, representing a more intense staining (*p* = 0.0342).

**Figure 5:**
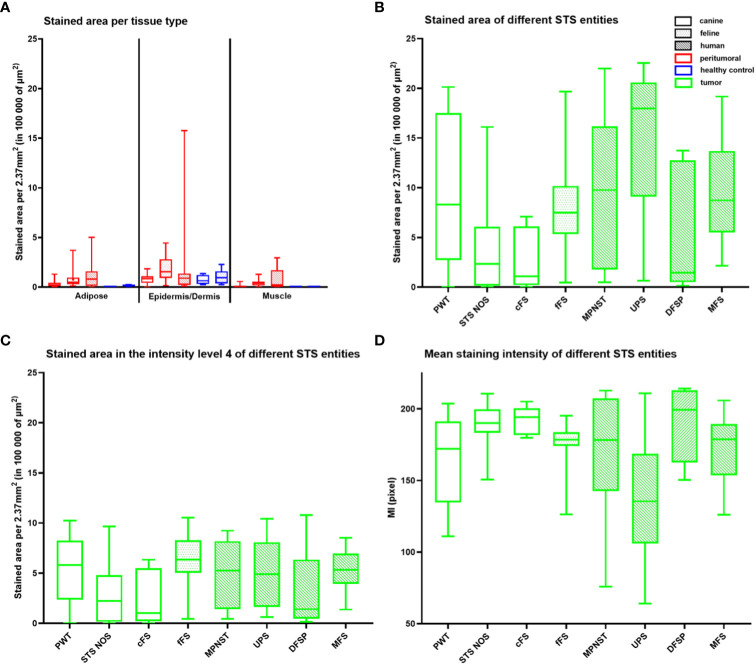
Quantitative analysis of the FAP staining in healthy (blue) and peritumoral (red) canine and feline tissue **(A)** and tumor tissue (green) of canine (unpatterned), feline (dotted pattern), and human STSs (striped pattern) **(B–D)**. **(A)** Box plot illustrating the FAP-stained area (µm^2^) per 10 high power fields (2.37 mm^2^) for the different tissue types—adipose tissue, epidermis/dermis, and muscle tissue—of the peritumoral and control tissue divided by species. FAP-stained areas in the peritumoral tissue of one tissue type are similar between species and similar if compared to the healthy control tissue of the same species (*p* > 0.2838). FAP-stained areas reach the highest values in the epidermis/dermis in all species with significant differences comparing peritumoral epidermis/dermis to peritumoral adipose tissue in dogs (*p* = 0.0043), peritumoral epidermis/dermis to peritumoral muscle tissue in dogs and cats (canine *p* = 0.0001, feline *p* = 0.0084), and healthy epidermis/dermis to healthy muscle tissue in dogs (dogs *p* = 0.0198). **(B–D)** Box plots representing the total stained area **(B)**, the stained area within level 4 (high staining intensity) **(C)**, and the mean staining intensity (MI) **(D)** of tumor tissue for the different STS entities. UPS has the largest stained area and the highest intensity of staining (low MI pixel value) with differences if compared to STS NOS (stained area *p* = 0.0045, MI *p* = 0.0267), cFS (stained area *p* = 0.0105), and DFSP (MI *p* = 0.0386). Stained areas **(C)** within level 4 differ for STS entities only between STS NOS and fFS (*p* = 0.0313). STS, soft tissue sarcoma; MPNST, malignant peripheral nerve sheath tumor; UPS, undifferentiated pleomorphic sarcoma; DFSP, dermatofibrosarcoma protuberans; MFS, myxofibrosarcoma; PWT, perivascular wall tumor; cFS, canine fibrosarcoma; STS NOS, STS not further specified; fFS, feline fibrosarcoma; FAP, fibroblast activation protein alpha.

**Figure 5A** illustrates the stained areas for the different tissue types (adipose tissue, epidermis/dermis, and muscle) of the peritumoral and control tissue samples. FAP-stained areas in the peritumoral tissue were similar between species and similar if compared to the healthy control tissue of the same species. FAP-stained areas reached the highest values in the dermis/epidermis in all species with significant differences comparing peritumoral epidermis/dermis to peritumoral adipose tissue in dogs (*p* = 0.0043) and peritumoral and healthy control epidermis/dermis compared to muscle tissue in dogs and cats (*p* < 0.0198).

Comparison of FAP labeling between STS entities detected differences in the stained tumor area between STS NOS and UPS (*p* = 0.0045) and cFS and UPS (*p* = 0.0105) (**Figure 5B**) with the largest stained tumor area in UPS. Splitting up the stained area into the staining intensity levels, level 4 was taken for comparison showing that there were no differences between STS entities independent of the species, with the only exception of a lower FAP staining in STS NOS compared to fFS (*p* = 0.0313) (**Figure 5C**). For the MI pixel values, differences were detected between STS NOS and UPS (*p* = 0.0267) and DFSP and UPS (*p* = 0.0386) (**Figure 5D**), with a higher intensity of FAP staining in UPS.

### Correlation analysis

There was a weak negative correlation between tumor grade and FAP-stained area (r = −0.3289, *p* < 0.0162) and a positive correlation with the MI pixel values (r = 0.3691, *p* = 0.0065) in dogs. Tumor grade did not correlate with the stained area in cats (r = 0.3473, *p* = 0.0964) or the MI pixel values (r = −0.2633, *p* = 0.2138).

There was a strong positive correlation between the visually assessed expression score and the FAP-stained area in canine and feline STSs (r = 0.8104, *p* < 0.0001) and in human STSs (r = 0.8272, *p* < 0.0001). A moderate negative correlation was found for the MI pixel values in feline and canine STSs (r = −0.6878, *p* < 0.0001) and a strong negative correlation for human STSs (r = −0.8511, *p* < 0.0001).

### Target selection criteria scoring

**Figure 6:**
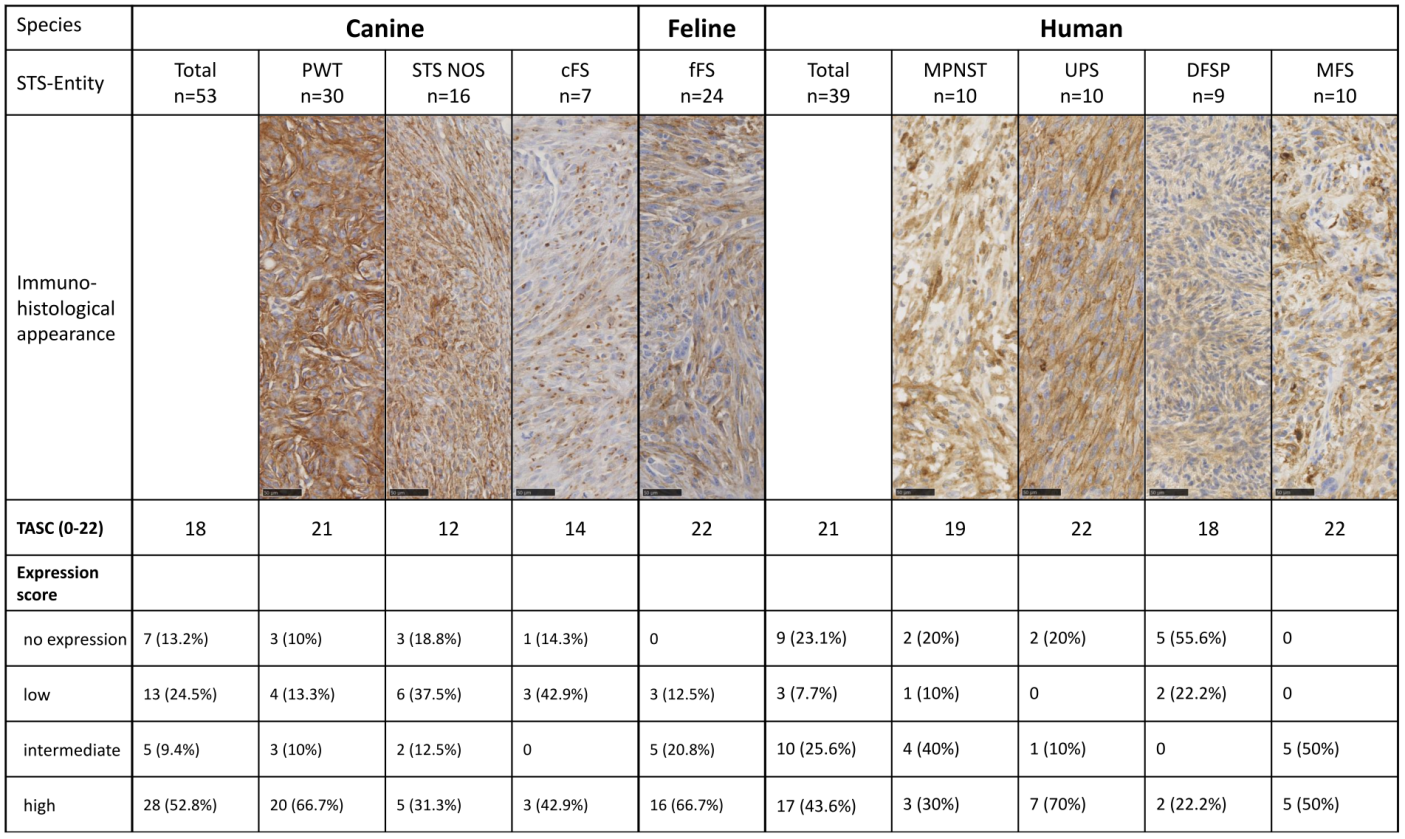
Target selection criteria (TASC) score and final FAP expression score for the different species and STS entities. STS, soft tissue sarcoma; MPNST, malignant peripheral nerve sheath tumor; UPS, undifferentiated pleomorphic sarcoma; DFSP, dermatofibrosarcoma protuberans; MFS, myxofibrosarcoma; PWT, perivascular wall tumor; cFS, canine fibrosarcoma; STS NOS, STS not further specified; fFS, feline fibrosarcoma; FAP, fibroblast activation protein alpha.

Diffuse overexpression throughout the tumor tissue (criterion II) defined as diffuse FAP staining in ≥50% of tumor cells in the majority (>50%) of the tumors was present in canine, feline, and human STSs and therefore achieved a maximal score of 4 (**Table 5**). In STS NOS and cFS, less than half of the tumors showed a FAP positivity over 50% of the tumor cells (score 0). A T/N ratio (criterion III) of over 10 could be reached in the majority of fFSs and human STSs (score 3), while less than half of the canine tumors had a ratio of over 10 (score 0). The lowest number of tumors with a T/N ratio over 10 was detected in MFS and STS NOS. The number of tumors with overexpression of FAP (criterion IV) was the highest in fFS (score 6) followed by human and canine STSs (score 5). Altogether, the TASC scoring of canine, feline, and human STSs was equal to or above 18 with the highest TASC score being achieved in fFS, indicating the suitability of FAP as the target for imaging in all species. The differentiation of TASC scores for the different STS entities is given in **Figure 6**. Only STS NOS and cFS displayed a TASC score below 18; all other entities showed scores above 18, classifying FAP as a suitable target for NIR imaging.

**Table 5:** Target selection criteria scoring for soft tissue sarcoma.

Species and STS entity	Criteria						TASC score (0–22)
	II		III		IV		
	Score	% tumors >50% pos. tumor cells	Score	Patients with T/N ratio >10	Score	% patients with overexpression*	
Human total	4	32/39 (82%)	3	18/34 (53%)	5	33/39 (85%)	21
MPNST	4	7/10 (90%)	0	3/9 (33%)	6	7/10 (90%)	19
UPS	4	9/10 (90%)	3	7/10 (70%)	6	9/10 (90%)	22
DFSP	4	7/9 (78%)	0	3/7 (43%)	5	7/9 (78%)	18
MFS	4	9/10 (90%)	3	5/8 (63%)	6	9/10 (90%)	22
Canine total	4	30/53 (57%)	0	20/44 (45%)	5	40/53 (76%)	18
PWT	4	22/30 (73%)	3	12/23 (52%)	5	25/30 (83%)	21
STS NOS	0	5/16 (32%)	0	5/14 (36%)	3	10/16 (62%)	12
cFS	0	3/7 (43%)	0	3/7 (43%)	5	5/7 (71%)	14
Feline fFS	4	19/24 (79%)	3	11/22 (50%)	6	22/24 (92%)	22

TASC, target selection criteria scoring; STS, soft tissue sarcoma; MPNST, malignant peripheral nerve sheath tumor; UPS, undifferentiated pleomorphic sarcoma; DFSP, dermatofibrosarcoma protuberans; MFS, myxofibrosarcoma; PWT, perivascular wall tumor; cFS, canine fibrosarcoma; STS NOS, STS not further specified; fFS, feline fibrosarcoma; FAP, fibroblast activation protein alpha. ^*^ % of patients with overexpression is defined as the number of FAP-positive tumors with more than 10% of the tumor cells expressing FAP.

## Discussion

In this immunohistochemical study, we describe the expression of FAP in selected human, canine, and feline STS subtypes using FFPE tissue samples. FAP expression was detected in 85% (33/39) of canine, 76% (40/53) of feline, and 92% (22/24) of human STSs. Quantitative immunohistological analyses confirm a significant overexpression of FAP in STSs compared to peritumoral, healthy skin, subcutaneous, and muscle tissue samples of the same species. Furthermore, we could prove similarities in the qualitative and quantitative IHC expression profiles of FAP in STS of humans and companion animals. Based on the results of this study and evidence extracted from the literature, FAP is a suitable and promising target for *in vivo* FGS of STS in humans, dogs, and cats.

*In vivo* optical FGS using targeted NIR fluorescent dyes is of great interest for intraoperative sarcoma imaging to ease complete tumor resection by visual guidance of the surgeon during the procedure. However, the heterogeneity and rarity of STS make target identification for FGS of these neoplasms especially challenging. Overexpression of the target structure in the neoplastic tissue samples is crucial to achieving a specific accumulation of targeted dyes in the tumor, and FAP is among the list of potential candidates.

FAP positivity was previously documented in human fibrosarcoma (5/5), MFS (4/4), leiomyosarcoma (8/10), liposarcoma (3/4), and undifferentiated sarcoma (2/3) cell lines (33). Dohi et al. (2008) demonstrated FAP positivity in frozen sections of three low-grade myofibroblastic sarcomas using IHC (34). In our study, 62.3% of canine (33/53), 87.5% of feline (21/24), and 69.2% of human STS (27/39) show an intermediate-to-high FAP expression score with 31%, 33%, and 42% of the tumor area being positive for FAP, respectively.

Clinically, radiolabeled FAPIs are promising for molecular imaging in people using PET/CT due to rapid blood clearance and limited uptake in normal and high uptake in tumor tissues, leading to excellent tumor-to-background ratios (24). Recently ^68^Ga-FAPI was used in a prospective clinical study for sarcoma imaging in 15 people. Its uptake intensity was positively correlated with IHC FAP expression (50). Furthermore, FAP-targeted radioligand therapy has been described in case reports including the first patients with STS (51, 52).

A second major limitation in the development of new treatment strategies for STS is the rarity of the condition in humans. The so-called rare cancers are hard to investigate, resulting in poor advances in diagnosis and treatment (53, 54). Therefore, in rare cancers, such as STS, the validation of suitable animal models is especially important.

Dogs are far more often affected by STS than humans (incidence 40:100,000) and represent an immunocompetent translational model of spontaneous carcinogenesis (3, 5). Due to their shorter lifespan and more rapid progression of cancer, trial completion is relatively fast and allows more timely assessment of clinical outcomes (55), which also applies to their feline counterpart. In addition, spontaneous companion animal models have several advantages over other animal trials such as exposition to the same environmental surrounding as people or spontaneous cancer development (55, 56). However, thorough basic research and more comparative studies are required to advance our understanding of where disease processes are similar or different across species. Despite dogs and cats being already advocated as ideal animal models for sarcoma research, direct cross-species comparative studies for target evaluation in STS are missing so far. We could show that significant FAP expression in STS is present in all three species, compared to the surrounding tissue. Based on these and other histological and IHC cross-species similarities, dogs and cats should be considered suitable translational models to further investigate FAP-targeted NIR dyes (2, 11, 56).

In addition to the overexpression in tumor tissue, the absence of target expression in non-neoplastic tissue samples is pivotal to achieving good contrast between the tumor and surrounding tissue. Previous investigations proposed an absence of low expression of FAP in several types of normal tissue samples (33, 34, 57, 58). Rettig et al. (1988) already observed marked FAP expression in scar tissue after surgical incisions but an absence of positivity in the adjacent dermis (33). In accordance with the described FAP expression in healing tissue, Dohi et al. (2008) proved high FAP expression in granulation tissue (34). The expression of FAP in wound healing, primarily in activated fibroblasts, is well known, while it is hardy restricted in normal fibroblasts (58). These findings are in alignment with our results: FAP-specific overexpression in the tumor samples compared to peritumoral and healthy control tissue samples was evident, while inflamed tissue samples show large areas of positive FAP labeling, diminishing the contrast. However, FAP expression in the inflamed peritumoral regions was significantly lower compared to inflammation tissue samples not associated with cancer (e.g., infection or injury). Therefore, we conclude that *in vivo* tumor margin delineation would still be feasible irrespective of peritumoral inflammatory reactions using an anti-FAP targeting NIR tracer. Taking into consideration these observations, FAP might not qualify as a suitable target in scar excision or revision of incomplete tumor resection shortly after the initial surgery due to the presence of activated fibroblasts in the healing wound.

Comparison of FAP expression among different STS entities is limited considering the small sample size within each STS entity cohort ranging between 7 and 30 tumor samples each. In addition, the classification of STS entities is far less precise in veterinary medicine compared to humans and is solely based on morphological features and IHC characterization (8). PWT, the largest cohort of included canine STSs, is composed of hemangiopericytomas, angioleiomyomas, myopericytomas, angiomyofibroblastomas, and angiofibromas (59). A subclassification of PWT is not routinely performed, as complex IHC panels are not routinely applied in veterinary pathology due to the associated high costs and the missing impact of subclassification on treatment (60). Common PWTs are hemangiopericytomas resembling their human counterpart (59) even though, in human prognosis, the metastatic rates and clinical characteristics of hemangiopericytoma patients vary greatly (61). PWT in dogs and UPS in humans were the entities with the highest FAP expression score, largest FAP-stained area, and highest intensity of staining in tumor tissue. In addition, those two entities showed the highest number of tumors with a T/N ratio over 10 if compared to the other entities of the same species, reaching the highest TASC scores together with MFS.

Human MFS and UPS have been screened for target expression previously. Tumor endothelial marker 1 (TEM1) and vascular endothelial growth factor A (VEGF A) were identified as the targets with the highest TASC scores (62). In addition, in MFS, TEM1 showed the highest tumor-to-background ratios assessed using IHC (63). VEGF A is also expressed in normal vasculature, and overexpression in the peritumoral tissue was detected in a few MFS cases (25). Lack of tumor specificity decreased its suitability for tumor targeting as reflected by the TASC score of 17 compared to 21 out of 22 for TEM1 (62) and FAP assessed in our study with 22 in human, 21 in feline, and 18 in canine STSs. Nevertheless, VEGF A has already been targeted during FGS in 15 human sarcoma patients using bevacizumab-800CW, proving its feasibility and safety (30). In dogs, VEGF expression is described in canine PWT (64), and recently, an IHC assessing overexpression of TEM1 in canine FS was published (65). Thus, their potential as targets for canine STS needs to be considered. However, all of those studies focused on specific STS entities, and no ideal target for the whole group of STS has been identified so far (38, 62).

Within a subgroup of STS, heterogeneity of FAP expression can be caused by factors such as the tumor grade or preoperative treatment. In human STS, we could not assess the impact of the tumor grade on the FAP expression statistically, as most of them were high grades. However, the investigated high-grade STS showed a high variability of FAP expression. In dogs, we found a non-significant negative correlation between higher tumor grade and FAP expression. If this finding is true and can be verified in independent studies, it would contradict findings in other tumor types where FAP overexpression is associated with an increase in the tumor’s aggressiveness. In canine mast cell tumors (40) as well as in human pancreatic adenocarcinoma, colon or lung cancer FAP is known to be a negative prognostic factor (66–68). In addition to high intertumoral heterogeneity, we found a marked intratumoral heterogeneity of FAP expression within single tumors. Whole-slide analysis as in this study better reflects this heterogeneity and limits sampling bias if compared to frequently used microarrays. Nevertheless, the analyzed slides still represent only a small fraction of the entire tumor. Intratumoral heterogeneity may be caused by genetically distinct cellular populations with certain subclonal mutations (69). Significant differences between tumor centers and margins were not investigated but could be of interest to future studies.

This study has several limitations. First, our results are solely based on the IHC of FFPE tissue samples, and no additional protein analyses such as Western blotting or PCR were included to confirm and further quantify the FAP expression. However, IHC staining of tumor tissue is a validated and commonly used technique to evaluate the degree of biomarker expression and has already been used for target screening in STS (25, 33, 62, 63). In contrast, there are currently no scores for imaging targets available based on PCR or sequencing data. Pre-analytic and post-analytic variables can influence IHC results (70) and especially the intensity of staining. As we used stored FFPE tissue samples of multiple institutions, pre-analytical variables such as the time point and duration of formaldehyde fixation, tissue handling during processing, or time of FFPE storage could not be controlled, leading to possible reaction bias (70, 71). Tissue collection, processing, and storage conditions can impair the immunoreactivity of the antigen epitopes. Analytical inaccuracy may result from differences in the staining protocol of human, canine, and feline tissue samples and different laboratories involved. In the initial pre-study phase, different antibodies were tested for their eligibility. Although polyclonal anti-FAP antibodies were previously utilized in canine mammary (72) and mast cell tumor tissue samples (40), in our study, a monoclonal anti-FAP primary antibody was selected, as it showed a more specific staining pattern. The previously described polyclonal antibody showed unspecific staining of epidermal and dermal tissue and high interspecies variation of background staining (own observations, not published). However, this problem is widely known; therefore, monoclonal antibodies are generally considered to be the more reliable choice (71). The specificity of the selected monoclonal antibody to bind FAP in dogs and cats was proven by Western blotting (**Supplementary Material**). Furthermore, the different antibody dilutions for staining of canine, feline, and human tissue were selected based on an optimal staining pattern depicted in control tissue after processing with antibody dilution series. The choice of diluent has a significant impact on the staining results, as the optimal antibody diluent supports antibody–epitope interactions and specificity. Due to this fact, we chose specific dilutions for each species, accepting that it may have an impact on the interspecies comparability of our results. However, there are several other factors such as masking of the epitopes by crosslinking of proteins after paraffin embedding, which need to be considered even after optimal antigen retrieval techniques are applied. We further tried to limit post-analytical variables and interpretation bias by using a uniform semiquantitative scoring system and artificial intelligence (AI) for quantitative image analysis.

Although our IHC results are promising, the *in vitro* expression of FAP does not necessarily reflect the *in vivo* expression of FAP on the surface of tumor cells in STS. Further *in vitro* studies are needed for the characterization and measurement of FAP expression of mesenchymal tumor cells. In addition, the effect of various neoadjuvant treatments on biomarker up- and downregulation needs to be studied in a larger cohort. Finally, we analyzed only a small fraction of STS entities with a relatively small sample size within each subgroup. Therefore, subgroup comparisons of FAP expression patterns need to be interpreted with caution. Nevertheless, this study represents the largest cohort of human STS samples investigated for the expression of FAP and is the first study investigating FAP expression in canine and feline STSs.

## Conclusion

Target specificity and tumor-specific uptake are critical determinants for the accuracy and efficacy of molecular NIR imaging. With this study on FFPE tissue samples, we could show that FAP is expressed in tumor cells in the majority of STSs of dogs, cats, and humans. Furthermore, our observations suggest that expression of FAP in healthy and peritumoral non-neoplastic tissues is limited to low levels, resulting in an adequate tumor-to-normal tissue ratio. These results underline that FAP is a possible target protein for NIR fluorescent imaging in STS and warrant further *in vitro*/*in vivo* studies to confirm these promising findings. In addition, results indicate that dogs and cats could serve as spontaneous large animal models for this rare but often fatal human disease.

## Data availability statement

The datasets supporting the conclusions of this article are freely and openly available at Harvard Dataverse, https://doi.org/10.7910/DVN/AX5090.

## Ethics statement

The studies involving humans were approved by Cantonal Ethics Commission Zurich (BASEC-2021-00417). The studies were conducted in accordance with local legislation and institutional requirements. Ethical review and approval were not required for the animal study because the use of stored canine and feline tissue samples initially taken for clinical purposes does not require ethical approval in Switzerland in accordance with the local legislation and institutional requirements. Written informed consent was not obtained from the owners for the participation of their animals in this study because the tissue used in this study derives from patients that underwent routine clinical treatment and was stored in veterinary pathology archives for diagnostic purposes. The inclusion of tissue samples was performed retrospectively.

## Author contributions

MN conceived of the presented idea. All authors contributed to the design and implementation of the research. Sample collection, processing, and qualitative analysis of canine and feline tissue samples were performed by PB, CK, and PG. Sample collection, processing, and qualitative analysis of human tissue samples were performed by MH and CP. Quantitative image analysis in all species was performed by PB and CK. PB analyzed the data and led the writing of the manuscript under the supervision of MN. The experiments for the validation of the cross-reactivity of the anti-FAP antibody in humans, dogs, and cats were planned, conducted, and interpreted by EB, DF, and EM. All authors approved the final manuscript.

## References

[1] von Mehren M Randall RL Benjamin RS Boles S Bui MM Ganjoo KN. Soft tissue sarcoma, version 2.2018, NCCN clinical practice guidelines in oncology. J Natl Compr Canc Netw (2018) 16(5):536–63. doi: 10.6004/jnccn.2018.0025 29752328

[2] Milovancev M Hauck M Keller C Stranahan LW Mansoor A Malarkey DE. Comparative pathology of canine soft tissue sarcomas: possible models of human non-rhabdomyosarcoma soft tissue sarcomas. J Comp Pathol (2015) 152(1):22–7. doi: 10.1016/j.jcpa.2014.09.005 25435513

[3] Stiller CA Trama A Serraino D Rossi S Navarro C Chirlaque MD. Descriptive epidemiology of sarcomas in Europe: report from the RARECARE project. Eur J Cancer (2013) 49(3):684–95. doi: 10.1016/j.ejca.2012.09.011 23079473

[4] Trama A Badalamenti G Baldi GG Brunello A Caira M Drove N. Soft tissue sarcoma in Italy: From epidemiological data to clinical networking to improve patient care and outcomes. Cancer Epidemiol (2019) 59:258–64. doi: 10.1016/j.canep.2019.02.012 30870746

[5] Graf R Pospischil A Guscetti F Meier D Welle M Dettwiler M. Cutaneous tumors in swiss dogs: retrospective data from the swiss canine cancer registry, 2008-2013. Vet Pathol (2018) 55(6):809–20. doi: 10.1177/0300985818789466 30131007

[6] Sbaraglia M Bellan E Dei Tos AP. The 2020 WHO Classification of Soft Tissue Tumours: news and perspectives. Pathologica (2021) 113(2):70–84. doi: 10.1016/j.canep.2019.02.012 33179614 PMC8167394

[7] Stiller CA Trama A Brewster DH Verne J Bouchardy C Navarro C. Descriptive epidemiology of Kaposi sarcoma in Europe. Report from the RARECARE project. Cancer Epidemiol (2014) 38(6):670–8. doi: 10.1016/j.ejca.2012.09.011 25454979

[8] Avallone G Boracchi P Stefanello D Ferrari R Rebughini A Roccabianca P. Canine perivascular wall tumors: high prognostic impact of site, depth, and completeness of margins. Veterinary Pathol (2014) 51(4):713–21. doi: 10.1177/0300985813503565 24048324

[9] Graf R Guscetti F Welle M Meier D Pospischil A. Feline injection site sarcomas: data from Switzerland 2009-2014. J Comp Pathol (2018) 163:1–5. doi: 10.1016/j.jcpa.2018.06.008 30213367

[10] Graf R Gruntzig K Boo G Hassig M Axhausen KW Fabrikant S. Swiss feline cancer registry 1965-2008: the influence of sex, breed and age on tumour types and tumour locations. J Comp Pathol (2016) 154(2-3):195–210. doi: 10.1016/j.jcpa.2016.01.008 26922257

[11] Gustafson DL Duval DL Regan DP Thamm DH. Canine sarcomas as a surrogate for the human disease. Pharmacol Ther (2018) 188:80–96. doi: 10.1016/j.pharmthera.2018.01.012 29378221 PMC6432917

[12] Chiti LE Ferrari R Roccabianca P Boracchi P Godizzi F Busca GA. Surgical margins in canine cutaneous soft-tissue sarcomas: A dichotomous classification system does not accurately predict the risk of local recurrence. Anim (Basel) (2021) 11(8):2367. doi: 10.3390/ani11082367 PMC8388623 34438827

[13] von Konow A Ghanei I Styring E Vult von Steyern F. Late local recurrence and metastasis in soft tissue sarcoma of the extremities and trunk wall: better outcome after treatment of late events compared with early. Ann Surg Oncol (2021) 28(12):7891–902. doi: 10.1245/s10434-021-09942-8 PMC8519908 33861406

[14] Sultan F Ganaie BA. Comparative oncology: Integrating human and veterinary medicine. Open Vet J (2018) 8(1):25–34. doi: 10.4314/ovj.v8i1.5 29445618 PMC5806664

[15] Vodanovich DA MC PF. Soft-tissue sarcomas. Indian J Orthop (2018) 52(1):35–44. doi: 10.4103/ortho.IJOrtho_220_17 29416168 PMC5791230

[16] Bray JP. Soft tissue sarcoma in the dog - part 1: a current review. J small Anim practice (2016) 57(10):510–9. doi: 10.1111/jsap.12556 27624929

[17] Dernell WS Withrow SJ Kuntz CA Powers BE. Principles of treatment for soft tissue sarcoma. Clin Tech Small Anim Pract (1998) 13(1):59–64. doi: 10.1016/S1096-2867(98)80029-7 9634350

[18] Ehrhart N. Soft-tissue sarcomas in dogs: a review. J Am Anim Hosp Assoc (2005) 41(4):241–6. doi: 10.5326/0410241 15995161

[19] Villedieu EJ Petite AF Godolphin JD Bacon NJ. Prevalence of pulmonary nodules suggestive of metastasis at presentation in dogs with cutaneous or subcutaneous soft tissue sarcoma. J Am Vet Med Assoc (2021) 258(2):179–85. doi: 10.2460/javma.258.2.179 33405989

[20] Müller N Kessler M. Curative-intent radical en bloc resection using a minimum of a 3 cm margin in feline injection-site sarcomas: a retrospective analysis of 131 cases. J Feline Med Surg (2018) 20(6):509–19. doi: 10.1177/1098612X17717882 PMC11104078 28696150

[21] Milovancev M Tuohy JL Townsend KL Irvin VL. Influence of surgical margin completeness on risk of local tumour recurrence in canine cutaneous and subcutaneous soft tissue sarcoma: A systematic review and meta-analysis. Vet Comp Oncol (2019) 17(3):354–64. doi: 10.1111/vco.12479 30953384

[22] Kainhofer V Smolle MA Szkandera J Liegl-Atzwanger B Maurer-Ertl W Gerger A. The width of resection margins influences local recurrence in soft tissue sarcoma patients. Eur J Surg Oncol (2016) 42(6):899–906. doi: 10.1016/j.ejso.2016.03.026 27107792

[23] Reyes Marlés RH Navarro Fernández JL Puertas García-Sandoval JP Santonja Medina F Mohamed Salem L Frutos Esteban L. Clinical value of baseline 18F-FDG PET/CT in soft tissue sarcomas. Eur J Hybrid Imaging (2021) 5(1):16. doi: 10.1186/s41824-021-00110-5 34476632 PMC8413431

[24] Koerber SA Finck R Dendl K Uhl M Lindner T Kratochwil C. Novel FAP ligands enable improved imaging contrast in sarcoma patients due to FAPI-PET/CT. Eur J Nucl Med Mol Imaging (2021) 48(12):3918–24. doi: 10.1007/s00259-021-05374-4 PMC8484190 34018010

[25] Rijs Z Shifai AN Bosma SE Kuppen PJK Vahrmeijer AL Keereweer S. Candidate biomarkers for specific intraoperative near-infrared imaging of soft tissue sarcomas: A systematic review. Cancers (Basel) (2021) 13(3):557. doi: 10.3390/cancers13030557 33535618 PMC7867119

[26] Hernot S van Manen L Debie P Mieog JSD Vahrmeijer AL. Latest developments in molecular tracers for fluorescence image-guided cancer surgery. Lancet Oncol (2019) 20(7):e354–e67. doi: 10.1016/s1470-2045(19)30317-1 31267970

[27] Bosma SE van Driel PB Hogendoorn PC Dijkstra PS Sier CF. Introducing fluorescence guided surgery into orthopedic oncology: A systematic review of candidate protein targets for Ewing sarcoma. J Surg Oncol (2018) 118(6):906–14. doi: 10.1038/s41467-018-05727-y PMC6220824 30212597

[28] Koller M Qiu SQ Linssen MD Jansen L Kelder W de Vries J. Implementation and benchmarking of a novel analytical framework to clinically evaluate tumor-specific fluorescent tracers. Nat Commun (2018) 9(1):3739. doi: 10.1038/s41467-018-05727-y 30228269 PMC6143516

[29] Pillozzi S Bernini A Palchetti I Crociani O Antonuzzo L Campanacci D. Soft tissue sarcoma: an insight on biomarkers at molecular, metabolic and cellular level. Cancers (Basel) (2021) 13(12):3044. doi: 10.3390/cancers13123044 34207243 PMC8233868

[30] Steinkamp PJ Pranger BK Li M-F Linssen MD Voskuil FJ Been LB. Fluorescence-guided visualization of soft-tissue sarcomas by targeting vascular endothelial growth factor A: A phase 1 single-center clinical trial. J Nucl Med (2021) 62(3):342–7. doi: 10.2967/jnumed.120.245696 32680922

[31] Hamson EJ Keane FM Tholen S Schilling O Gorrell MD. Understanding fibroblast activation protein (FAP): substrates, activities, expression and targeting for cancer therapy. Proteomics Clin Appl (2014) 8(5-6):454–63. doi: 10.1002/prca.201300095 24470260

[32] Fitzgerald AA Weiner LM. The role of fibroblast activation protein in health and Malignancy. Cancer Metastasis Rev (2020) 39(3):783–803. doi: 10.1007/s10555-020-09909-3 32601975 PMC7487063

[33] Rettig WJ Garin-Chesa P Beresford HR Oettgen HF Melamed MR Old LJ. Cell-surface glycoproteins of human sarcomas: differential expression in normal and Malignant tissues and cultured cells. Proc Natl Acad Sci U S A (1988) 85(9):3110–4. doi: 10.1073/pnas.85.9.3110 PMC280153 2896356

[34] Dohi O Ohtani H Hatori M Sato E Hosaka M Nagura H. Histogenesis-specific expression of fibroblast activation protein and dipeptidylpeptidase-IV in human bone and soft tissue tumours. Histopathology (2009) 55(4):432–40. doi: 10.1111/j.1365-2559.2009.03399.x PMC2784039 19817894

[35] Liu F Qi L Liu B Liu J Zhang H Che D. Fibroblast activation protein overexpression and clinical implications in solid tumors: a meta-analysis. PloS One (2015) 10(3):e0116683. doi: 10.1371/journal.pone.0116683 25775399 PMC4361589

[36] Lindner T Giesel FL Kratochwil C Serfling SE. Radioligands targeting fibroblast activation protein (FAP). Cancers (Basel) (2021) 13(22):5744. doi: 10.3390/cancers13225744 34830898 PMC8616197

[37] de Geus SW Boogerd LS Swijnenburg R-J Mieog JSD Tummers WS Prevoo HA. Selecting tumor-specific molecular targets in pancreatic adenocarcinoma: paving the way for image-guided pancreatic surgery. Mol Imaging Biol (2016) 18(6):807–19. doi: 10.1073/pnas.85.9.3110 PMC5093212 27130234

[38] van Oosten M Crane LM Bart J van Leeuwen FW van Dam GM. Selecting potential targetable biomarkers for imaging purposes in colorectal cancer using TArget Selection Criteria (TASC): a novel target identification tool. Trans Oncol (2011) 4(2):71–82. doi: 10.1593/tlo.10220 PMC3069650 21461170

[39] Roccobianca P Schulman Y Avallone G Foster R Scruggs J Dittmer K. Pathology of tumors of domestic animals. In: Tumors of soft tissue, vol. 3. Davis Thompson Foundation, Formoor Ln Gurnee, IL, USA (2020).

[40] Giuliano A Dos Santos Horta R Constantino-Casas F Hoather T Dobson J. Expression of fibroblast activating protein and correlation with histological grade, mitotic index and ki67 expression in canine mast cell tumours. J Comp Pathol (2017) 156(1):14–20. doi: 10.1016/j.jcpa.2016.10.004 27889201

[41] Meuten D. Appendix: diagnostic schemes and algorithms. In: DJ M, editor. Tumors in domestic animals, John Wiley & Sons, Inc. (2016). p. 942–78.

[42] Gu B Liu X Wang S Xu X Hu S Yan W. Head-to-head evaluation of [^18^F]FDG and [^68^ Ga]Ga-DOTA-FAPI-04 PET/CT in recurrent soft tissue sarcoma. Eur J Nucl Med Mol Imaging (2022) 49:2889–2901. doi: 10.1007/s00259-022-05700-4 PMC9206606 35113192

[43] Kratochwil C Flechsig P Lindner T Abderrahim L Altmann A Mier W. Ga-FAPI PET/CT: tracer uptake in 28 different kinds of cancer. J Nucl Med (2019) 60(6):801–5. doi: 10.1007/s00259-021-05273-8 PMC6581228 30954939

[44] Li J Chen K Liu H Cheng K Yang M Zhang J. Activatable near-infrared fluorescent probe for *in vivo* imaging of fibroblast activation protein-alpha. Bioconjug Chem (2012) 23(8):1704–11. doi: 10.1021/bc300278r PMC3419799 22812530

[45] Hintz HM Gallant JP Vander Griend DJ Coleman IM Nelson PS LeBeau AM. Imaging fibroblast activation protein alpha improves diagnosis of metastatic prostate cancer with positron emission tomography. Clin Cancer Res (2020) 26(18):4882–91. doi: 10.1158/1078-0432.CCR-20-1358 PMC7683011 32636317

[46] Roy J Hettiarachchi SU Kaake M Mukkamala R Low PS. Design and validation of fibroblast activation protein alpha targeted imaging and therapeutic agents. Theranostics (2020) 10(13):5778–89. doi: 10.7150/thno.41409 PMC7254991 32483418

[47] Loktev A Lindner T Burger EM Altmann A Giesel F Kratochwil C. Development of fibroblast activation protein-targeted radiotracers with improved tumor retention. J Nucl Med (2019) 60(10):1421–9. doi: 10.2967/jnumed.118.224469 PMC6785792 30850501

[48] Hu K Li J Wang L Huang Y Li L Ye S. Preclinical evaluation and pilot clinical study of [^18^F]AlF-labeled FAPI-tracer for PET imaging of cancer associated fibroblasts. Acta Pharm Sin B (2022) 12(2):867–75. doi: 10.1016/j.apsb.2021.09.032 PMC8897030 35256951

[49] Fischer E Chaitanya K Wüest T Wadle A Scott AM van den Broek M. Radioimmunotherapy of fibroblast activation protein positive tumors by rapidly internalizing antibodies. Clin Cancer Res (2012) 18(22):6208–18. doi: 10.1158/1078-0432.CCR-12-0644 22992515

[50] Kessler L Ferdinandus J Hirmas N Bauer S Dirksen U Zarrad F. Ga-FAPI as a diagnostic tool in sarcoma: data from the. J Nucl Med (2022) 63(1):89–95. doi: 10.2967/jnumed.121.262096 33931468 PMC8717183

[51] Kratochwil C Giesel FL Rathke H Fink R Dendl K Debus J. [(153)Sm]Samarium-labeled FAPI-46 radioligand therapy in a patient with lung metastases of a sarcoma. Eur J Nucl Med Mol Imaging (2021) 48(9):3011–3. doi: 10.1007/s00259-021-05273-8 PMC8263436 33728499

[52] Ferdinandus J Costa PF Kessler L Weber M Hirmas N Kostbade K. Initial clinical experience with. J Nucl Med (2022) 63(5):727–34. doi: 10.2967/jnumed.121.262468 PMC9051597 34385340

[53] Casali PG Trama A. Rationale of the rare cancer list: a consensus paper from the Joint Action on Rare Cancers (JARC) of the European Union (EU). ESMO Open (2020) 5(2):e000666. doi: 10.1136/esmoopen-2019-000666 32220947 PMC7174011

[54] Gatta G van der Zwan JM Casali PG Siesling S Dei Tos AP Kunkler I. Rare cancers are not so rare: the rare cancer burden in Europe. Eur J Cancer (2011) 47(17):2493–511. doi: 10.1016/j.ejca.2011.08.008 22033323

[55] Garden OA Volk SW Mason NJ Perry JA. Companion animals in comparative oncology: One Medicine in action. Vet J (2018) 240:6–13. doi: 10.1016/j.tvjl.2018.08.008 30268334

[56] Klosowski M Haines L Alfino L McMellen A Leibowitz M Regan D. Naturally occurring canine sarcomas: Bridging the gap from mouse models to human patients through cross-disciplinary research partnerships. Front Oncol (2023) 13:1130215. doi: 10.3389/fonc.2023.1130215 37035209 PMC10076632

[57] Schuberth PC Hagedorn C Jensen SM Gulati P van den Broek M Mischo A. Treatment of Malignant pleural mesothelioma by fibroblast activation protein-specific re-directed T cells. J Transl Med (2013) 11:187. doi: 10.1186/1479-5876-11-187 23937772 PMC3751305

[58] Ebert LM Yu W Gargett T Toubia J Kollis PM Tea MN. Endothelial, pericyte and tumor cell expression in glioblastoma identifies fibroblast activation protein (FAP) as an excellent target for immunotherapy. Clin Transl Immunol (2020) 9(10):e1191. doi: 10.1002/cti2.1191 PMC7557106 33082953

[59] Avallone G Helmbold P Caniatti M Stefanello D Nayak RC Roccabianca P. The spectrum of canine cutaneous perivascular wall tumors: morphologic, phenotypic and clinical characterization. Veterinary Pathol (2007) 44(5):607–20. doi: 10.1354/vp.44-5-607 17846233

[60] Avallone G Stefanello D Ferrari R Roccabianca P. The controversial histologic classification of canine subcutaneous whorling tumours: The path to perivascular wall tumours. Vet Comp Oncol (2020) 18(1):3–8. doi: 10.1111/vco.12559 31778274

[61] Wang K Mei F Wu S Tan Z. Hemangiopericytoma: incidence, treatment, and prognosis analysis based on SEER database. BioMed Res Int (2020) 2020:2468320. doi: 10.1155/2020/2468320 33204688 PMC7655240

[62] de Gooyer JM Versleijen-Jonkers YMH Hillebrandt-Roeffen MHS Frielink C Desar IME de Wilt JHW. Immunohistochemical selection of biomarkers for tumor-targeted image-guided surgery of myxofibrosarcoma. Sci Rep (2020) 10(1):2915. doi: 10.1038/s41598-020-59735-4 32076024 PMC7031512

[63] Rijs Z Belt E Kalisvaart GM Sier CFM Kuppen PJK Cleven AHG. Immunohistochemical evaluation of candidate biomarkers for fluorescence-guided surgery of myxofibrosarcoma using an objective scoring method. Biomedicines (2023) 11(3):982. doi: 10.3390/biomedicines11030982 36979961 PMC10046284

[64] Avallone G Stefanello D Boracchi P Ferrari R Gelain ME Turin L. Growth factors and COX2 expression in canine perivascular wall tumors. Vet Pathol (2015) 52(6):1034–40. doi: 10.1177/0300985815575050 25795373

[65] Marzec M Kandefer-Gola M Janus I Bubak J Nowak M. Endosialin (CD248) expression in fibromas and soft-tissue fibrosarcomas in dogs. In Vivo (2021) 35(3):1467–72. doi: 10.21873/invivo.12399 PMC8193292 33910824

[66] Patsouras D Papaxoinis K Kostakis A Safioleas MC Lazaris AC Nicolopoulou-Stamati P. Fibroblast activation protein and its prognostic significance in correlation with vascular endothelial growth factor in pancreatic adenocarcinoma. Mol Med Rep (2015) 11(6):4585–90. doi: 10.3892/mmr.2015.3259 25625587

[67] Wikberg ML Edin S Lundberg IV Van Guelpen B Dahlin AM Rutegård J. High intratumoral expression of fibroblast activation protein (FAP) in colon cancer is associated with poorer patient prognosis. Tumour Biol (2013) 34(2):1013–20. doi: 10.1007/s13277-012-0638-2 PMC3597266 23328994

[68] Moreno-Ruiz P Corvigno S Te Grootenhuis NC La Fleur L Backman M Strell C. Stromal FAP is an independent poor prognosis marker in non-small cell lung adenocarcinoma and associated with p53 mutation. Lung Cancer (2021) 155:10–9. doi: 10.1016/j.lungcan.2021.02.028 33706022

[69] Dentro SC Leshchiner I Haase K Tarabichi M Wintersinger J Deshwar AG. Characterizing genetic intra-tumor heterogeneity across 2,658 human cancer genomes. Cell (2021) 184(8):2239–54.e39. doi: 10.1016/j.cell.2021.03.009 33831375 PMC8054914

[70] Fedchenko N Reifenrath J. Different approaches for interpretation and reporting of immunohistochemistry analysis results in the bone tissue - a review. Diagn Pathol (2014) 9:221. doi: 10.1186/s13000-014-0221-9 25432701 PMC4260254

[71] Matos LL Trufelli DC de Matos MG da Silva Pinhal MA. Immunohistochemistry as an important tool in biomarkers detection and clinical practice. biomark Insights (2010) 5:9–20. doi: 10.4137/bmi.s2185 20212918 PMC2832341

[72] Ettlin J Clementi E Amini P Malbon A Markkanen E. Analysis of gene expression signatures in cancer-associated stroma from canine mammary tumours reveals molecular homology to human breast carcinomas. Int J Mol Sci (2017) 18(5):1101. doi: 10.3390/ijms18051101 28531107 PMC5455009

