# Supplementary material for: Cross-species evaluation of fibroblast activation protein alpha as potential imaging target for soft tissue sarcoma: a comparative immunohistochemical study in humans, dogs, and cats
Source: Front Oncol. 2023 Sep 1;13:1210004. doi: 10.3389/fonc.2023.1210004 (PMC10505752; doi:10.3389/fonc.2023.1210004)
Supplement: Supplementary file 1 [file DataSheet_1.docx]

Supplementary Material

Antibody validation

**Patricia Beer^1*^, Chantal Pauli^2,3^, Martina Haberecker^2^, Paula Grest^4^, Erin Beebe^5^, Daniel Fuchs^5^, Enni Markkanen^5^, Christiane Krudewig^4^†, Mirja Christine Nolff^1^†**

^1^Clinic for Small Animal Surgery, Department for Small Animals, Vetsuisse Faculty, University of Zurich, Zurich, Switzerland

^2^Department of Pathology and Molecular Pathology, University Hospital Zurich, Zurich, Switzerland

^3^Medical Faculty, University of Zurich, Zurich, Switzerland

^4^Institute of Veterinary Pathology, Vetsuisse Faculty, University of Zurich, Zurich, Switzerland

^5^Institute of Veterinary Pharmacology and Toxicology, Vetsuisse Faculty, University of Zurich, Zurich, Switzerland

*** Correspondence:**Patricia Beer
[pbeer@vetclinics.uzh.ch](mailto:pbeer@vetclinics.uzh.ch)

† These authors contributed equally to this work and share senior authorship

# Materials and Methods

**Cells and cell lines**

BJ5ta human fibroblasts were purchased from ATCC. FSII cells derived from feline fibrosarcoma as originally described in ^1^, and DUS cells are derived from canine uterine stroma, as detailed in ^2,3^. Cells of Dog 6 and Dog 10 are primary cell lines derived from two PWT patient tumors. To isolate cells from these tumors, freshly excised tissue from surgery was collected in falcon tubes with sterile PBS, washed 3 times with PBS containing 100 U/ml penicillin (Gibco) and 100 μg/ml streptomycin (Gibco) and cut into small pieces using sterile scalpel blades. The tissue was then resuspended in 10 ml DMEM with 60 μg/ml Liberase Blendzyme 3 (Roche) and 1x Antibiotic Antimycotic Solution (Sigma-Aldrich) and incubated at 37°C for 30 – 90 min until digestion was complete. Digestion was stopped by addition of 30 ml of warm DMEM with 15% FBS (Gibco) and 1 x Antibiotic Antimycotic solution and cells were pelleted by centrifugation at 520 g for 5 min. The cell pellet was washed 2 x by resuspending in 40 ml warm DMEM with 15% FBS and 1 x Antibiotic Antimycotic solution, followed by 5 min centrifugation @ 500g. Subsequently, the pellet was resuspended in 10 ml warm DMEM with 15% FBS and 1 x Antibiotic Antimycotic solution, transferred to a 10 cm cell culture dish and placed in an incubator @37°C with 5% CO_2_. Plates were checked daily for tumor cell outgrowth for the next days, medium was changed when necessary, and cells were split for freezing and passaging when reaching the confluency of 80 – 90%. For regular cell culture, BJ5ta were cultured in 1 x DMEM + 10% FBS at @37°C with 5% CO_2_. All canine and feline cells were cultured in 1 x DMEM (Gibco) with high glucose (4.5 g/L), supplemented with 1x Non-essential Amino Acids (Gibco), 1 x Antibiotic Antimycotic Solution and 15% FBS.

**qRT-PCR**

Total RNA was purified using the RNeasy® Mini Kit by QIAGEN according to the manufacturer’s protocol. Equal amounts of RNA were reverse transcribed using the BioRad iScript™ cDNA Synthesis Kit according to the manufacturer’s protocol with the LabCycler (SensoQuest). Quantitative reverse transcription PCR (qRT-PCR) was performed using the KAPA SYBR® FAST One-Step qRT-PCR Kit in a total volume of 10 μl in duplicates on the CFX384 Touch™ Real-Time PCR detection system (Bio-Rad) using following primers from Microsynth (Balgach, Switzerland): cFAP-fw 5’-TTTGGAGTTGCCACCTCTGC-3’, cFAP-rev 5’-TGTTGTACCTCCTTCGGAGTC-3’, cGAPDH-fw 5’- CATCACTGCCACCCAGAAG-3’, cGAPDH-rev 5’- CAGTGAGCTTCCCGTTCAG-3’, cB2M-fw 5’- TCCTCATCCTCCTCGCT-3’, cB2M-rev 5’-TTCTCTGCTGGGTGTCG-3’. Quantification of gene expression was performed using the comparative CT method, values were normalized against GAPDH and B2M, and DUS cells as the control, and results were expressed as fold change in mRNA levels over DUS cells. Each experiment was independently repeated two times as indicated in the figure legends, and data are expressed as individual data points and mean ± SD.

**Western blot**

For Western blotting, whole cell extracts that were prepared from exponentially growing cells using the RIPA lysis buffer and protein concentration was measured using the Pierce^TM^ BCA Protein Assay Kit (thermos Scientific, USA). For each sample, 40 μg of total protein extract was separated on 4-20% gradient gels (BioRad) and transferred onto nitrocellulose 0.2µm membranes (BioRad) using the Transfer-Blot Turbo Transfer System (BioRad) according to the manufacturer’s instructions. Blots were blocked overnight with 5 % nonfat dry milk in TBST at 4°C, and subsequently probed with primary antibodies against FAP (ab207178, 1:325, 2 h @ RT, Abcam) and Tubulin (T5168, 1:10’000, 1 h @RT, Sigma), and secondary HRP-linked antibodies against rabbit (A0545, 1:5’000, 1 h @RT, Sigma) and mouse (#A2304, 1:10000, 1 h @RT, Sigma). Detection and quantification were performed using the SuperSignal West Femto or Pico Chemiluminescent substrate (ThermoScientific, USA) using the Chemidoc Imaging System (Biorad). Tubulin served as the loading control.

**Immunohistochemistry**

Immunohistochemistry (IHC) of formalin fixated and paraffin embedded tumor tissue of DOG 6 and DOG 10 was performed as described in the main manuscript using the anti-FAP alpha recombinant monoclonal rabbit antibody (ab207178; EPR20021; Abcam).

# Results

Western blot analysis shows target specificity of anti-FAP alpha recombinant monoclonal rabbit antibody (ab207178) with a signal for FAP at the expected molecular weight of 95kDA in all three species (Supplemental Fig. 1). Absence of signal in dog uterine stroma cells compared to human fibroblasts, canine soft tissue sarcoma cells and feline fibrosarcoma cells confirms that the antibody is specific for FAP, as DUS cells show very low expression of FAP, as confirmed by qPCR (Suppl. Fig 1B).

IHC of the FFPE tissue of DOG 6 and 10 is displayed in Supplemental Figure 2 showing a high expression of FAP within the tumor.

## Supplementary Figure 1


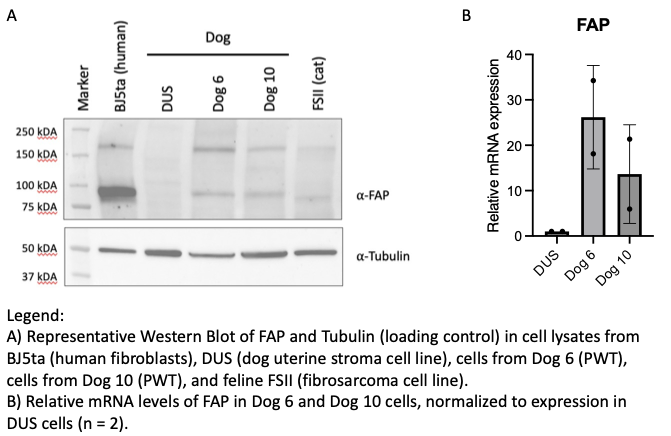


**Supplementary Figure 1.** A) Representative Western blot image of FAP (top panel) and Tubulin (lower panel, loading control) in cell lysates from BJ5ta (human fibroblasts), DUS (dog uterine stroma cell line), primary patient-derived tumor cells from Dog 6 (PWT) and Dog 10 (PWT), and feline FSII (fibrosarcoma cell line). B) Relative mRNA levels of FAP in Dog 6 and Dog 10 cells, normalized to expression in DUS cells (n=2).

## Supplementary Figure 2

**Supplementary Figure 2.** Images of immunohistochemically stained FFPE tumor tissue using anti-FAP alpha recombinant monoclonal rabbit antibody (ab207178B) in A) Dog 6 and B) Dog 10. Both perivascular wall tumors show a high FAP expression in the tumor compared to the peritumoral tissue (top images magnification 0,4x). The tumor cells are positive for FAP (bottom magnification 40x).

# References

1. von Erichsen, J., Hecht, W., Löhberg-Gruene, C. & Reinacher, M. Cell Lines Derived From Feline Fibrosarcoma Display Unstable Chromosomal Aneuploidy and Additionally Centrosome Number Aberrations. Vet Pathol. 2012;49(4):648–657. https://doi.org:10.1177/0300985811406887
2. Guscetti F, Nassiri S, Beebe E, Rito Brandao I, Graf R, Markkanen E. et al. Molecular homology between canine spontaneous oral squamous cell carcinomas and human head-and-neck squamous cell carcinomas reveals disease drivers and therapeutic vulnerabilities. Neoplasia (United States). 2020;22(12):778-788. https://doi.org:10.1016/j.neo.2020.10.003
3. Graubner FR, Reichler IM, Rahman NA, Payan-Carreira R, Boos A, Kowalewski MP. Decidualization of the canine uterus: From early until late gestational in vivo morphological observations, and functional characterization of immortalized canine uterine stromal cell lines. Reproduction in Domestic Animals. 2017;137–147. https://doi.org:10.1111/rda.12849
